# Astroglial tau pathology alone preferentially concentrates at sulcal depths in chronic traumatic encephalopathy neuropathologic change

**DOI:** 10.1093/braincomms/fcaa210

**Published:** 2020-12-03

**Authors:** John D Arena, Victoria E Johnson, Edward B Lee, Garrett S Gibbons, Douglas H Smith, John Q Trojanowski, William Stewart

**Affiliations:** 1 Department of Neurosurgery, Penn Center for Brain Injury and Repair, Perelman School of Medicine, University of Pennsylvania, Philadelphia, PA 19104, USA; 2 Department of Pathology and Laboratory Medicine, Center for Neurodegenerative Disease Research, Perelman School of Medicine, University of Pennsylvania, Philadelphia, PA 19104, USA; 3 Translational Neuropathology Research Laboratory, University of Pennsylvania, Philadelphia, PA 19104, USA; 4 Department of Neuropathology, Queen Elizabeth University Hospital, Glasgow G51 4TF, UK; 5 Institute of Neuroscience and Psychology, University of Glasgow, Glasgow G12 8QQ, UK

**Keywords:** chronic traumatic encephalopathy, Alzheimer’s disease, tau, ageing-related tau astrogliopathy, traumatic brain injury

## Abstract

Current diagnostic criteria for the neuropathological evaluation of the traumatic brain injury-associated neurodegeneration, chronic traumatic encephalopathy, define the pathognomonic lesion as hyperphosphorylated tau-immunoreactive neuronal and astroglial profiles in a patchy cortical distribution, clustered around small vessels and showing preferential localization to the depths of sulci. However, despite adoption into diagnostic criteria, there has been no formal assessment of the cortical distribution of the specific cellular components defining chronic traumatic encephalopathy neuropathologic change. To address this, we performed comprehensive mapping of hyperphosphorylated tau-immunoreactive neurofibrillary tangles and thorn-shaped astrocytes contributing to chronic traumatic encephalopathy neuropathologic change. From the Glasgow Traumatic Brain Injury Archive and the University of Pennsylvania Center for Neurodegenerative Disease Research Brain Bank, material was selected from patients with known chronic traumatic encephalopathy neuropathologic change, either following exposure to repetitive mild (athletes *n* = 17; non-athletes *n* = 1) or to single moderate or severe traumatic brain injury (*n* = 4), together with material from patients with previously confirmed Alzheimer’s disease neuropathologic changes (*n* = 6) and no known exposure to traumatic brain injury. Representative sections were stained for hyperphosphorylated or Alzheimer’s disease conformation-selective tau, after which stereotypical neurofibrillary tangles and thorn-shaped astrocytes were identified and mapped. Thorn-shaped astrocytes in chronic traumatic encephalopathy neuropathologic change were preferentially distributed towards sulcal depths [sulcal depth to gyral crest ratio of thorn-shaped astrocytes 12.84 ± 15.47 (mean ± standard deviation)], with this pathology more evident in material from patients with a history of survival from non-sport injury than those exposed to sport-associated traumatic brain injury (*P* = 0.009). In contrast, neurofibrillary tangles in chronic traumatic encephalopathy neuropathologic change showed a more uniform distribution across the cortex in sections stained for either hyperphosphorylated (sulcal depth to gyral crest ratio of neurofibrillary tangles 1.40 ± 0.74) or Alzheimer’s disease conformation tau (sulcal depth to gyral crest ratio 1.64 ± 1.05), which was comparable to that seen in material from patients with known Alzheimer’s disease neuropathologic changes (*P* = 0.82 and *P* = 0.91, respectively). Our data demonstrate that in chronic traumatic encephalopathy neuropathologic change the astroglial component alone shows preferential distribution to the depths of cortical sulci. In contrast, the neuronal pathology of chronic traumatic encephalopathy neuropathologic change is distributed more uniformly from gyral crest to sulcal depth and echoes that of Alzheimer’s disease. These observations provide new insight into the neuropathological features of chronic traumatic encephalopathy that distinguish it from other tau pathologies and suggest that current diagnostic criteria should perhaps be reviewed and refined.

## Teaser

Mapping hyper-phosphorylated tau pathologies contributing to the pathognomonic cortical lesion of chronic traumatic encephalopathy neuropathologic change reveals immunoreactive astrocytes preferentially concentrate to sulcal depths. In contrast, neurofibrillary tangles show limited sulcal concentration, comparable with Alzheimer’s disease. These data provide new insight into the specific pathology of chronic traumatic encephalopathy.

## Graphical Abstract

**Graphical Abstract:**
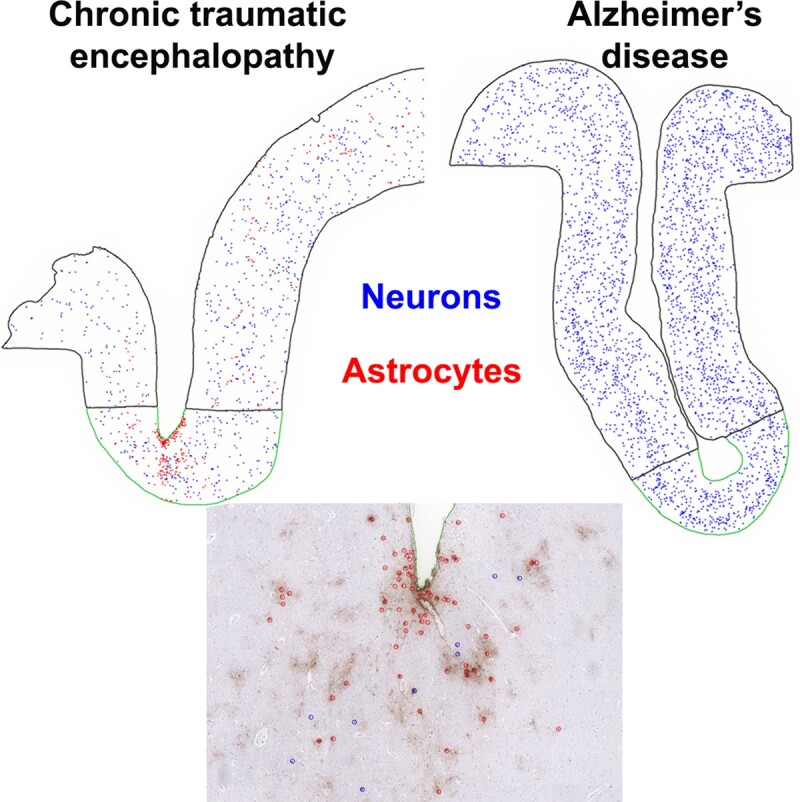

## Introduction

Traumatic brain injury (TBI) is recognized as a major risk factor for a range of neurodegenerative diseases, including Alzheimer’s disease and chronic traumatic encephalopathy (CTE) (Johnson *et al.*, 2017; Smith *et al.*, 2019). Although first described, several decades ago, as dementia pugilistica of former boxers (Millspaugh, 1937; Critchley, 1949; Critchley, 1957; Corsellis *et al.*, 1973), consensus criteria for the neuropathological assessment of what is now recognized as CTE only emerged in the last decade (McKee *et al.*, 2016). These criteria define the pathognomonic lesion of CTE neuropathologic change (CTE-NC) as *p-tau aggregates in neurons, astrocytes and cell processes around small vessels in an irregular pattern at the depths of cortical sulci* (McKee *et al.*, 2016). However, despite incorporation into diagnostic criteria, the neocortical features currently defining CTE-NC have not been formally evaluated using rigorous and objective measures.

Earliest accounts of the neuropathology of CTE (then dementia pugilistica) described abundant neuronal profiles in the form of neurofibrillary tangles, with a noted preferential involvement of superficial cortical layers and patchy distribution of pathology. Also documented in these early reports was the subjective impression of an apparent concentration of tangles to the depths of cortical sulci (Hof *et al.*, 1992; Geddes *et al.*, 1996; Geddes *et al.*, 1999), although this was never formally quantified. In addition to neurofibrillary tangle pathology, tau-immunoreactive, thorn-shaped astrocytes (TSAs) are increasingly recognized as a prominent feature of CTE (Ikeda *et al.*, 1995; Ikeda *et al.*, 1998; McKee *et al.*, 2009; McKee *et al.*, 2013; McKee *et al.*, 2016), with the combination of these glial and neuronal pathologies defining the pathognomonic lesion of CTE-NC (McKee *et al.*, 2016); a pathology that has now been documented not only in boxers, but in non-boxer athletes (Omalu *et al.*, 2005; McKee *et al.*, 2009; McKee *et al.*, 2013; Smith *et al.*, 2013; Stewart *et al.*, 2016; Ling *et al.*, 2017; Mez *et al.*, 2017; Lee *et al.*, 2019), military veterans (Goldstein *et al.*, 2012; Stein *et al.*, 2014) and individuals exposed to a single moderate or severe TBI (Johnson *et al.*, 2012; Zanier *et al.*, 2018; Tribett *et al.*, 2019; Arena *et al.*, 2020).

While neurofibrillary tangles have long been described in association with a variety of neurodegenerative pathologies, only more recently attention has been paid to astrocytic tau pathologies in ageing and wider neurodegeneration. Ageing-related tau astrogliopathy (ARTAG) is increasingly recognized in the brains of older individuals and as a co-morbidity in a variety of neurodegenerative diseases (Kovacs *et al.*, 2016; Kovacs *et al.*, 2017), with its presence suggested to correlate with a greater degree of cognitive impairment (Robinson *et al.*, 2018). Comprising morphologically distinct phospho-tau immunoreactive astrocytes, ARTAG typically localizes to interface sites, such as sub-pial, perivascular or sub-ependymal locations, or around the boundary between grey and white matter (Kovacs *et al.*, 2016). Notably, one morphological variant of ARTAG, TSA, shows comparable morphology and immunophenotype to the astroglial pathology of CTE-NC (Kovacs *et al.*, 2016; Liu *et al.*, 2016; Kovacs *et al.*, 2017; Kovacs *et al.*, 2018; Forrest *et al.*, 2019; Arena *et al.*, 2020).

There is, therefore, a need to critically appraise the pathology of CTE-NC and, where required, refine neuropathological criteria for its recognition and differentiation from the pathologies of ageing and wider neurodegenerative disease. To this end, we pursued quantitative mapping of the distribution of cortical neurofibrillary tangle and astroglial pathologies in cases with previously documented CTE-NC and in cases with Alzheimer’s disease neuropathologic changes and no known history of TBI. Contrary to understanding reflected in current diagnostic criteria, our observations demonstrate that the astroglial pathology alone shows marked concentration to the depths of cortical sulci in CTE-NC. In contrast, neurofibrillary tangles show only limited concentration towards sulcal depths, which echoes that seen in Alzheimer’s disease.

## Materials and methods

All tissue samples were obtained from the Glasgow TBI Archive, Department of Neuropathology, Queen Elizabeth University Hospital, Glasgow, UK, or the University of Pennsylvania Center for Neurodegenerative Disease Research (CNDR) Brain Bank, Philadelphia, PA, USA. Brain tissue was acquired by means of planned donation after multi-year longitudinal follow-up, or at routine diagnostic autopsy. Ethical approval for use of tissue in this study was provided by the West of Scotland Research Ethics Committee (Project ID 225271), the Greater Glasgow and Clyde Biorepository (Application Number 340) and the Institutional Review Board of the University of Pennsylvania.

Cases for inclusion were identified as all available cases from each archive with documented pathology of CTE-NC, or a random sample of cases from the CNDR archive with known Alzheimer’s disease neuropathologic changes and no known history of exposure to TBI. All CTE-NC cases (*n* = 22) had a history of previous exposure to head trauma, had been subject to comprehensive and standardized neuropathological assessment for neurodegenerative disease pathologies at autopsy and had documented cortical tau pathology consistent with the pathognomonic pathology of CTE-NC defined in diagnostic criteria (McKee *et al.*, 2016). In each case where CTE-NC was present, the extent and distribution of pathology was further dichotomized as ‘low’ or ‘high’; corresponding to stages I/II or III/IV, respectively, of a widely used protocol (McKee *et al.*, 2013). For CTE-NC cases, histories included a remote history of exposure to repetitive mild TBI (soccer, *n* = 8; American football, *n* = 4; rugby union, *n* = 3; boxing, *n* = 1; mixed sports, *n* = 1; non-sport, *n* = 1) or a single moderate or severe TBI (fall, *n* = 2; assault, *n* = 1; motor vehicle collision, *n* = 1). Detailed reports from the original diagnostic autopsies were available for all cases; supplemented, where necessary, by forensic and clinical records. All Alzheimer’s disease cases had no documented history of TBI or participation in contact sport, and met neuropathological criteria for the diagnosis of Alzheimer’s disease [*n* = 6; 4 high and 2 intermediate Alzhemier’s disease neuropathologic changes (ADNC) (Montine *et al.*, 2012)]. A single representative cortical sulcus containing pathognomonic hyperphosphorylated tau (p-tau) pathology was selected for analysis in each case. Regionally matched tissue sections from the frontal, angular and temporal cortices were selected from ADNC cases for comparison with CTE-NC. Clinical, demographic and neuropathologic information for each case, including integrated clinicopathologic diagnosis (Lee, 2018; Lee *et al.*, 2019), is summarized in Table 1.

**Table 1:** Case demographics

Case no.	Age at death	Sex	TBI/sport exposure	Integrated clinicopathologic diagnosis	CTE-NC stage	PMI
C1	40s	M	Football	CBD	Low	7 h
C2	50s	M	sTBI, Fall	No NDD	Low	108 h
C3	60s	M	Football	CBD	Low	3 h
C4	60s	M	Multiple non-sport mTBI	DLB	Low	21 h
C5	70s	M	Football	DLB	Low	18 h
C6	70s	M	Rugby	AD	Low	48 h
C7	70s	M	Rugby	Mixed AD/VaD	Low	48 h
C8	70s	M	sTBI, MVC	CTE	Low	24 h
C9	70s	M	sTBI, Fall	PDD	Low	7.5 h
C10	80s	M	Soccer	AD	Low	24 h
C11	80s	M	Soccer	AD	Low	20 h
C12	50s	M	Soccer	CTE	High	Unknown
C13	60s	M	sTBI, Assault	No NDD	High	24 h
C14	60s	M	Boxing	CTE	High	24 h
C15	60s	M	Soccer	VaD	High	11 days
C16	70s	M	Rugby	CTE	High	12 h
C17	70s	M	Soccer	DLB	High	4 days
C18	70s	M	Soccer	CTE	High	3 days
C19	80s	M	Football	FTLD-TDP	High	7 h
C20	80s	M	Soccer	NPH	High	3 days
C21	80s	M	Soccer	CTE and PD	High	3 days
C22	80s	M	Boxing, Rugby, Soccer	PDD	High	4 days
A1	60s	M	No	AD	n/a	13.5 h
A2	60s	M	No	AD	n/a	5 h
A3	70s	M	No	AD	n/a	4 h
A4	70s	M	No	AD	n/a	8.5 h
A5	70s	F	No	AD	n/a	11 h
A6	80s	F	No	AD	n/a	6 h

AD, Alzheimer’s disease; ADNC, Alzheimer’s disease neuropathologic changes; CBD, corticobasal degeneration; CTE-NC, chronic traumatic encephalopathy neuropathologic change; DLB, dementia with Lewy bodies; FTLD-TDP, frontotemporal lobar degeneration with TDP-43 inclusions; MVC, motor vehicle collision; n/a, not applicable; NDD, neurodegenerative disease; NPH, normal pressure hydrocephalus; PD, Parkinson’s disease; PDD, Parkinson’s disease dementia; PMI, post-mortem interval; sTBI, single moderate or severe traumatic brain injury; TBI, traumatic brain injury; VaD, vascular dementia.

### Brain tissue handling and immunohistochemistry

Whole brains from the Glasgow TBI Archive were fixed in 10% formol saline at autopsy for a minimum of 2 weeks prior to dissection, standardized anatomical sampling, tissue processing and paraffin embedding as described previously (Graham *et al.*, 1995). Tissue blocks sampled from fresh brains at the University of Pennsylvania CNDR were fixed overnight in 70% ethanol and 150 mmol sodium chloride or 10% neutral-buffered formalin and processed to paraffin as described previously (Toledo *et al.*, 2014). For each case, a single representative cortical block was chosen to include the defining pathognomonic pathology of CTE-NC or, for AD cases, a high burden of ADNC. From each tissue block, 8 μm tissue sections were prepared and subjected to deparaffinization and rehydration to H_2_O before immersion in 3% aqueous H_2_O_2_ to quench endogenous peroxidase activity. Antigen retrieval was performed via microwave pressure cooker in either Tris/EDTA (CP13) or citrate buffer following formic acid pre-treatment (GT-38), as optimized for each antibody. Sections were blocked using normal horse serum (Vector Labs, Burlingame, CA) in Optimax buffer (BioGenex, Fremont, CA) for 30 min followed by incubation in the primary antibody overnight at 4°C. Specifically, tau antibodies CP13 (specific for phosphoepitope S202, 1:1000 dilution, courtesy Dr Peter Davies) (Jicha *et al.*, 1999) and GT-38 (1:1000 dilution, UPenn CNDR) were applied. GT-38 has been shown to detect a conformation-dependent epitope present in tau within the inclusions of Alzheimer’s disease requiring both 3R and 4R tau, but not the 3R or 4R-only tau of other primary tauopathies, and in a phosphorylation-independent manner (Gibbons *et al.*, 2018; Gibbons *et al.*, 2019). After rinsing, sections were incubated in a biotinylated universal secondary antibody (Vector Labs) for 30 min, followed by the avidin–biotin complex for 30 min (Vector Labs). Visualization was achieved using the DAB peroxidase substrate kit (Vector Labs). Sections were counterstained with haematoxylin, followed by rinsing, dehydration and coverslipping. Sections from a known positive control were stained in parallel with test sections, with omission of the primary antibody for one section to control for non-specific staining.

### Mapping of pathologies

Stained sections from the Glasgow TBI Archive were scanned at a magnification of 20× with a Hamamatsu NanoZoomer 2.0-HT slide scanner, saved as NDPI files and evaluated using Aperio ImageScope Viewer 12.3.3 software (Leica Biosystems, Wetzlar, Germany). Sections from the University of Pennsylvania CNDR were scanned at a magnification of 20× with a Lamina Slide Scanner (Perkin Elmer, Waltham, MA) and saved as MSRX files, exported to ImageJ (National Institutes of Health, Bethesda, MD) using QuPath Open Source Digital Pathology software and then saved as TIFF file format and evaluated using Aperio ImageScope Viewer 12.3.3 (Leica Biosystems, Wetzlar, Germany).

**Figure 1:**
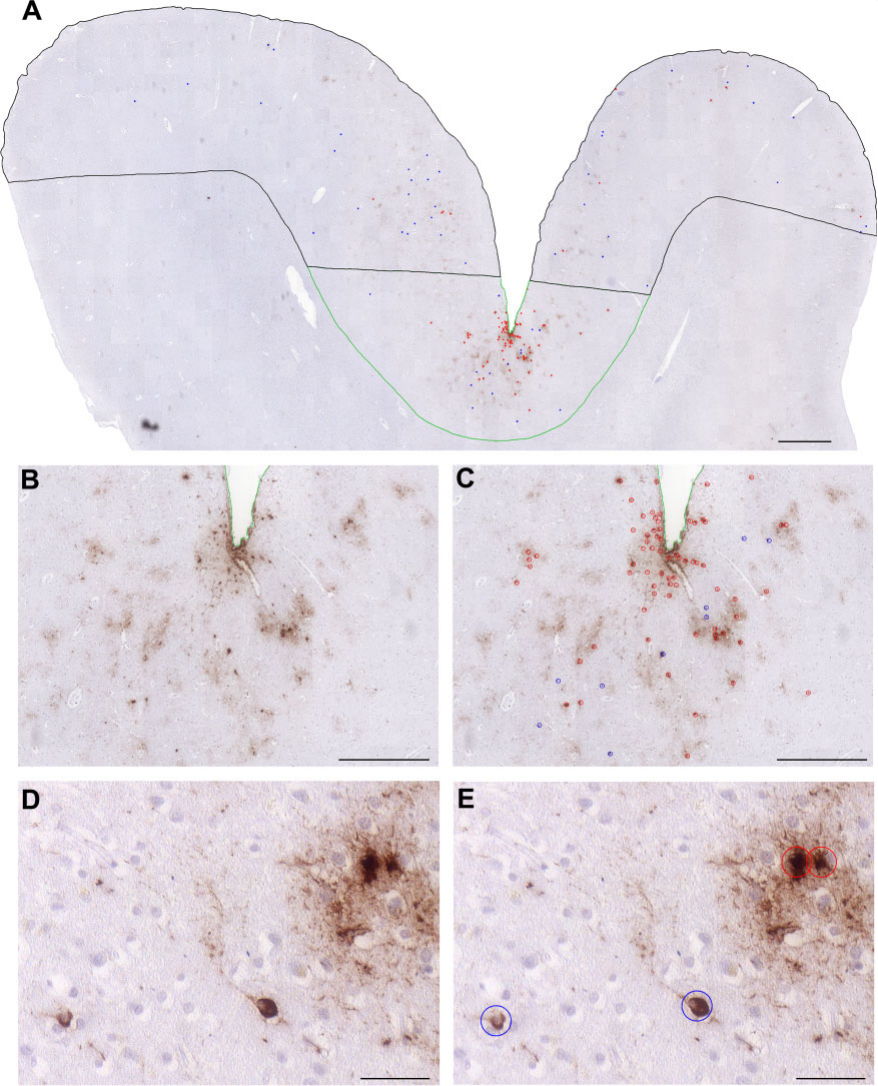
**Neuronal and astroglial tau pathologies of CTE neuropathologic change.** (**A**) Low power view of cortical material from a former American football player who died in his 70s with a diagnosis of dementia with Lewy bodies (Case C5) reveals a patchy distribution of p-tau-immunoreactive (CP13) profiles, with apparent concentration of pathology to the sulcal depth (outlined in green) compared to the gyral crest (outlined in black). (**B, C**) There is clustering of p-tau immunoreactive profiles around cortical vessels, (**D, E**) which on higher power are revealed as typical neurofibrillary tangles (blue circles) and thorn-shaped astrocytes (red circles). Scale bars: (**A**) 1 mm, (**B, C**) 500 μm and (**D, E**) 50 μm

On each scanned image, the region of interest was defined as the cortical grey matter of an entire involved sulcus and the adjacent gyral crests in which the pathologies of either CTE-NC or ADNC were present. This region of interest was then further sub-divided into sulcal depth and gyral crest (non-depth cortex) regions; the sulcal depth region defined as extending 1 mm from the deepest aspect of the sulcus towards the gyral crest and to the underlying grey-white junction (Holleran *et al.*, 2017). In accordance with standard diagnostic and research practices, neuronal or astroglial tau-immunoreactive pathologies were identified as classical neurofibrillary tangles or thorn-shaped astrocytes by their defining and stereotypical cellular morphologies (Montine *et al.*, 2012; Crary *et al.*, 2014; Kovacs, 2015; Kovacs *et al.*, 2016; McKee *et al.*, 2016; Kovacs *et al.*, 2017). CP13 immunostaining revealed both neurofibrillary tangles and thorn-shaped astrocytes, whereas GT-38 exclusively identified neurofibrillary tangles, as described previously (Arena *et al.*, 2020). The location of each immunoreactive profile was then annotated within the region of interest (Fig. 1). All assessments were performed blind to clinical information and neuropathological diagnoses.

### Quantification of pathology and statistical analysis

Densities of tau-immunoreactive profiles in each region were calculated as the number of profiles per mm^2^ assessed. From these data, a depth to crest ratio was calculated for neurofibrillary tangles or thorn-shaped astrocytes by dividing the density of the pathology in the depth sub-region by the density in the adjacent crest. A subset of cortical regions was independently annotated and assessed by two reviewers, resulting in a good-to-excellent inter-rater reliability, with intra-class correlation coefficient 0.923 (95% confidence interval, 0.718–0.980; two-way random effects, absolute agreement and single measurement) (Koo and Li, 2016).

### Statistical analyses

Statistical analyses were performed using STATA v15.1 statistics software (College Station, TX) and GraphPad Prism v8.2.1 (San Diego, CA). Non-parametric tests were used in comparisons of depth to crest ratio, as assumptions of normality were not met after tests of skewness and kurtosis. Unpaired data samples were compared using the Mann–Whitney test, and paired samples were compared using the Wilcoxon signed-rank test. Associations between pathology and age were calculated by linear regression. Statistical significance was determined using an alpha level of 0.05.

### Data availability

The authors confirm that the data supporting the findings of this study are available within the article.

## Results

### Thorn-shaped astrocytes show concentration at sulcal depths in CTE-neuropathologic change

**Figure 2:**
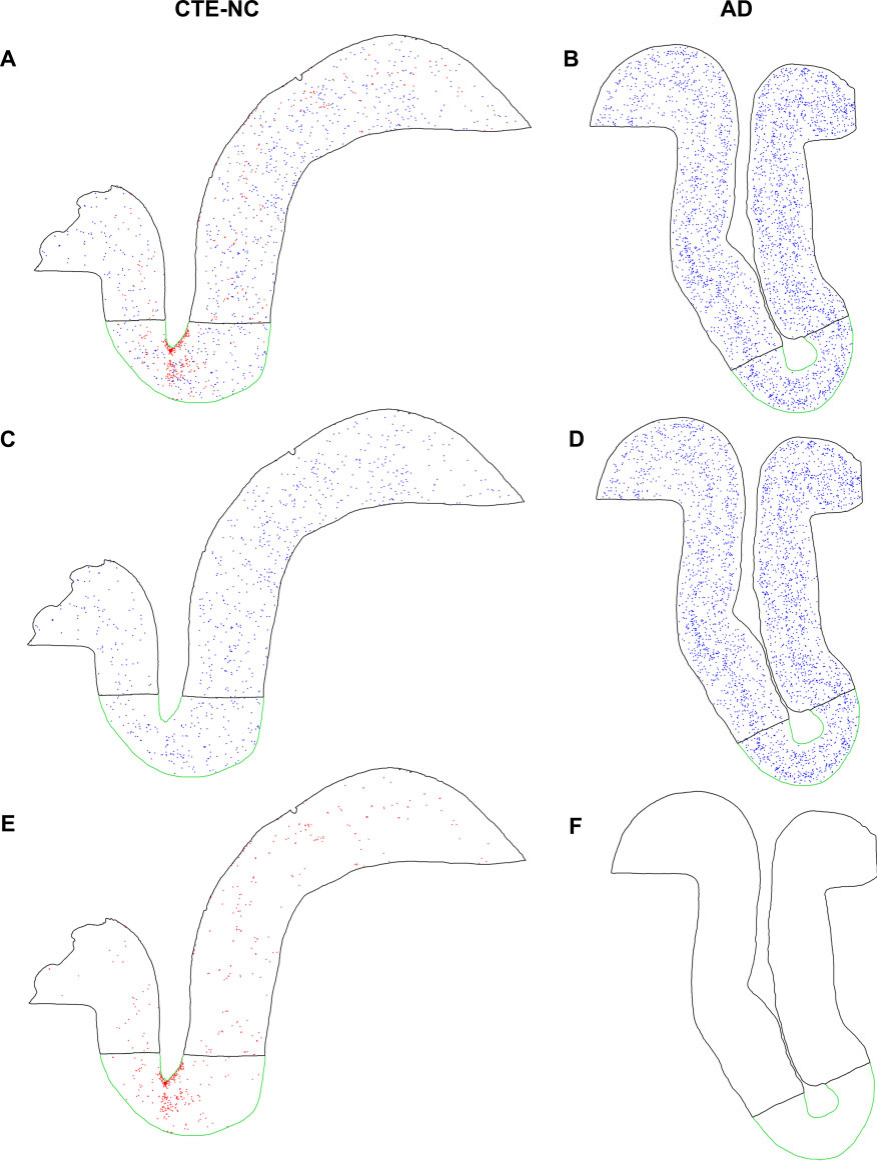
**Maps of neurofibrillary tangles and thorn-shaped astrocytes in CTE-NC and ADNC.** (**A**) Reviewing the annotated map of p-tau-immunoreactive (CP13) profiles in CTE-NC reveals numerous neurofibrillary tangles (blue) and thorn-shaped astrocytes (red), (**C**) with the former showing relatively uniform distribution across the sulcal depth (outlined in green) and gyral crest (outlined in black). (**E**) In contrast, thorn-shaped astrocytes (red) show marked clustering and concentration to the sulcal depth. (**B**) In parallel, analysis of ADNC (**D**) reveals a heavy burden of neurofibrillary tangles arranged in a classical bilaminar distribution across the cortex, with limited concentration to the sulcal depth. (**F**) No thorn-shaped astrocytes were present in ADNC. (**A, C, E**) Case C10, a former soccer player age 80s. (**B, D, F**) Case A6, a patient with known Alzheimer’s disease (age, 80 years)

**Figure 3:**
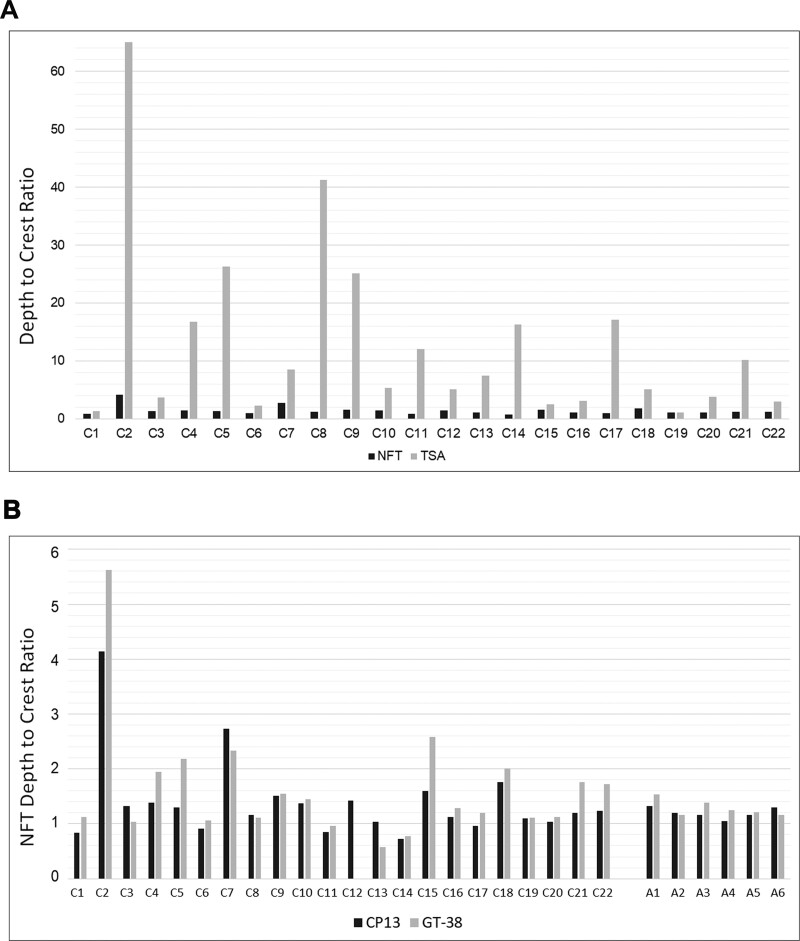
**Sulcal depth to gyral crest distributions of neuronal and astroglial pathologies in each case.** (**A**) In cases with known CTE-NC, the ratio of sulcal depth to gyral crest CP13-positive thorn-shaped astrocyte pathology was typically high, in contrast to neurofibrillary tangles, which showed only limited sulcal concentration. (**B**) There was a close correlation between density of neurofibrillary tangles immunoreactive for CP13 and neuronal profiles identified using the antibody GT-38, which detects a conformation-dependent epitope of tau present in Alzheimer’s disease. The sulcal depth concentration of these pathologies in cases with CTE-NC was similar to that seen in cases with ADNC. NFT, neurofibrillary tangles; TSA, thorn-shaped astrocytes

**Figure 4:**
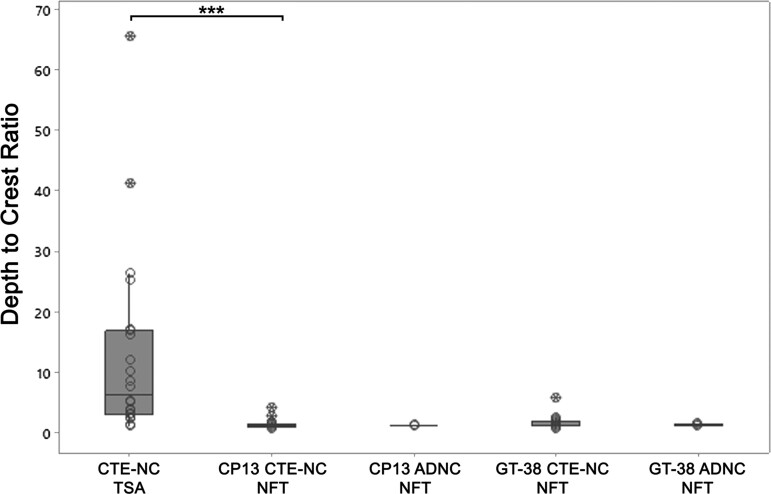
**Depth to crest ratio of tau pathologies in CTE-NC and ADNC.** Quantitative assessment of thorn-shaped astrocytes and neuronal profiles in each case reveals astrocytes with a higher ratio of sulcal depth to gyral crest density (sulcal depth to gyral crest ratio 12.84 ± 15.47; mean ± SD) than co-existing neurofibrillary tangles (1.40 ± 0.74; *P* < 0.001). There was mild concentration of both CP13- and GT-38-immunoreactive neurofibrillary tangles in CTE-NC, which was similar to that seen in material from patients with ADNC (all analyses not significant). TSA, thorn-shaped astrocytes; NFT, neurofibrillary tangles; open circles, individual data points; crossed circles, outliers

In keeping with previous descriptions, low power examination of sections from cases with known CTE-NC showed a patchy deposition of p-tau immunoreactivity, with apparent concentration to the depths of cortical sulci and distribution to superficial cortical layers (Fig. 1). While p-tau-immunoreactive thorn-shaped astrocytes were not observed in sections with ADNC, these were abundant in cases with CTE-NC (Fig. 1). Typically, p-tau immunoreactive thorn-shaped astrocytes in cases with CTE-NC were present in sub-pial locations and extending into deeper cortical layers (Fig. 2), with clustering around intracortical vessels. Formal mapping and quantitative assessment revealed thorn-shaped astrocytes in CTE-NC heavily concentrated to the sulcal depths (sulcal depth to gyral crest ratio of thorn-shaped astrocytes 12.84 ± 15.47, mean ± SD) (Figs. 3A and 4).

### Limited sulcal neurofibrillary tangle concentration in CTE-neuropathologic change echoes Alzheimer’s disease

Mapping of neuronal profiles demonstrated a mild increase in density of p-tau immunoreactive (CP13) neurofibrillary tangles and GT-38 immunoreactive profiles at the depths of cortical sulci compared to adjacent gyral crests in cases with CTE-NC and also in cases with ADNC (Figs. 2 and 3B). Notably, the mild sulcal depth concentration of neuronal profiles in CTE-NC was similar to that observed in cases with ADNC, whether assessed in sections stained for CP13 [sulcal depth to gyral crest ratio of neurofibrillary tangles in CTE-NC 1.40 ± 0.74 (mean ± standard deviation); ADNC 1.20 ± 0.10; *P* = 0.82; Mann–Whitney test] or for GT-38 (1.64 ± 1.05, CTE-NC; 1.29 ± 0.15, ADNC; *P* = 0.91) (Fig. 4). Furthermore, the sulcal depth to gyral crest concentration of neurofibrillary tangles in CTE-NC was considerably lower than that of thorn-shaped astrocytes in matched sections (*P* < 0.001, Wilcoxon signed-rank test) (Fig. 4).

### Association between injury and patient variables and distribution of thorn-shaped astrocytes in CTE

**Figure 5:**
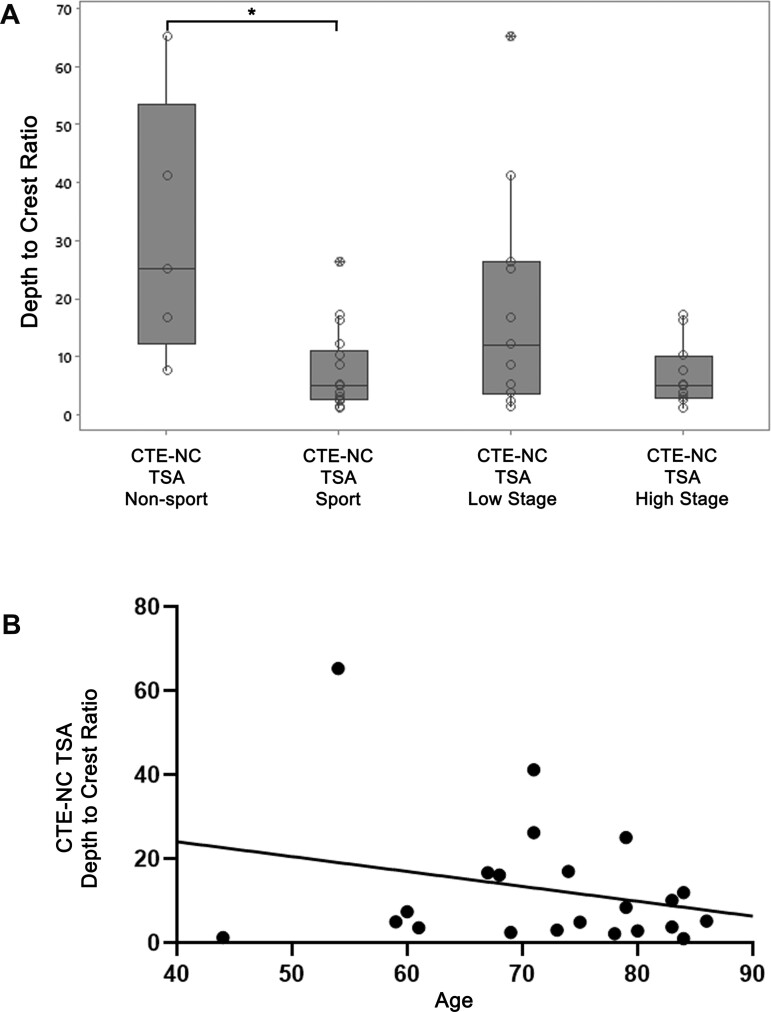
**Association of injury exposure, CTE-NC stage and age at death with thorn-shaped astrocyte distribution.** (**A**) Concentration of thorn-shaped astrocytes to the sulcal depth was greater where there was a history of non-sports TBI (depth to crest ratio, 31.21 ± 22.79) than when exposure to TBI was through participation in contact sports (7.44 ± 6.92: *P* = 0.009). Although there was a greater concentration of thorn-shaped astrocytes to sulcal depths in cases with low (18.90 ± 19.80) versus high (6.78 ± 5.48) stage CTE-NC, this was not significant (*P* = 0.12). (**B**) There was no association between age at death and distribution of thorn-shaped astrocytes (*R*^2^ = 0.063, *P* = 0.26). TSA, thorn-shaped astrocytes; open circles, individual data points; crossed circles, outliers

Sub-dividing cases with known CTE-NC by history of injury exposure demonstrated greater sulcal concentration of thorn-shaped astrocytes in cases with a history of survival from non-sport TBI than in cases with history of exposure to repetitive mild TBI through contact sports participation (sulcal depth to gyral crest ratio of thorn-shaped astrocytes 31.21 ± 22.79 versus 7.44 ± 6.92; *P* = 0.009; Fig. 5A). No difference in concentration of CP13 or GT-38 neurofibrillary tangles was noted between sport and non-sport TBI cases. There was no significant difference in sulcal concentration of thorn-shaped astrocytes in low-stage compared to high-stage CTE-NC (18.90 ± 19.80 versus 6.78 ± 5.48; p = 0.12) (Fig. 5A). Sucal depth to gyral crest ratios of both neurofibrillary tangles and thorn-shaped astrocytes (Fig. 5B) was independent of patient age.

## Discussion

Herein, we present formal, quantitative evaluation of the distribution of neocortical neuronal and astroglial tau pathologies contributing to CTE neuropathologic change. Specifically, our data demonstrate that the typical p-tau immunoreactive, thorn-shaped astrocytes of CTE-NC showed considerable concentration to the depths of cortical sulci compared to adjacent gyral crests. In contrast, p-tau immunoreactive neurofibrillary tangles showed only limited concentration at the depths of cortical sulci in CTE-NC, which was not different from that observed in material from patients with known Alzheimer’s disease neuropathologic changes. Notably, the concentration of p-tau-immunoreactive astroglial pathology to the depths of cortical sulci in CTE-NC was greater in material from patients with a history of survival from non-sport TBI than in cases with a history of exposure to repetitive mild TBI through participation in contact sports. In contrast, we found no clear association between cortical distribution of these pathologies and neuropathological stage of CTE-NC or with patients’ age at death.

Prior studies have assessed the distribution of individual components of p-tau pathology in CTE-NC (Armstrong *et al.*, 2017, 2019). In contrast to our data demonstrating that the astroglial pathologies alone show distinctive concentration to the depths of cortical sulci, previous quantitative analysis suggested both astrocytic and neuronal pathologies concentrated to the sulcal depths (Armstrong *et al.*, 2017). However, unlike this study, no comparison with equivalent cortical p-tau pathologies in wider neurodegenerative disease, including Alzheimer’s disease, was pursued. In this context, we observed sulcal concentration of neurofibrillary tangles in CTE-NC when compared to gyral crests; however, this was mild in comparison to the equivalent assessment of astroglial pathology. Furthermore, the mild concentration of neurofibrillary tangles we observed in cases with CTE-NC was of the same degree as that recorded in our cases with ADNC which, in turn, was consistent with the previous quantitative assessments of cortical distribution of pathologies in ADNC (Gentleman *et al.*, 1992; Clinton *et al.*, 1993; Armstrong, 1994). Preferential distribution of neurofibrillary tangle pathology to sulcal depths, therefore, appears neither a marked nor a specific finding in CTE-NC.

Although finding only limited concentration of neurofibrillary tangle pathology at sulcal depths in CTE-NC, thorn-shaped astrocytes showed marked sulcal concentration. Early descriptions of thorn-shaped astrocytes in ARTAG reported distribution to the depths of cortical sulci in the temporal lobe (Ikeda *et al.*, 1995; Ikeda, 1996). Furthermore, reported patterns of subpial ARTAG across the cerebral convexities share a striking resemblance to CTE-NC and, in part, are hypothesized might result from prior exposure to trauma (Kovacs *et al.*, 2018). Elsewhere, re-evaluation of Corsellis’ original cohort revealed ARTAG pathology in 10 of 14 ex-boxers, including all cases with sulcal CTE-NC (Goldfinger *et al.*, 2018). Sub-pial and perivascular thorn-shaped astrocytes have also been described in chronic survivors of military blast injury (Shively *et al.*, 2016) and spinal cord thorn-shaped astrocytes develop in the setting of chronic compressive forces in cervical spondylosis (Shimizu *et al.*, 2008). Therefore, the possibility exists that, in the context of neurodegeneration, distribution of astroglial tau pathology towards sulcal depths might serve as evidence of prior exposure to TBI (Forrest *et al.*, 2019).

Prior characterization has revealed that the neuronal and astroglial pathologies of CTE-NC contain differing tau isoform compositions and immunophenotypes, echoing tau phenotypes of comparable pathologies in ageing and Alzheimer’s disease (Arena *et al.*, 2020). As such, in addition to shared cellular morphologies and anatomic distributions, thorn-shaped astrocytes of CTE-NC and ARTAG are composed of 4R tau, with similar post-translational modifications (Arena *et al.*, 2020). Furthermore, assessment of the distribution of ARTAG has implicated a possible role for chronic disruption of CSF–brain and blood–brain barriers in its development (Kovacs *et al.*, 2017; Kovacs *et al.*, 2018). In this context, persisting and widespread blood–brain barrier disruption has been shown following both single moderate or severe (Hay *et al.*, 2015) and repetitive mild TBI in humans (Doherty *et al.*, 2016), and as a pathological consequence of mild TBI in experimental models (Johnson *et al.*, 2018). As such, the possibility that the astroglial pathologies of CTE-NC and ARTAG share similar aetiologies might be considered.

Current consensus criteria for the neuropathological assessment and diagnosis of CTE define the pathognomonic lesion based on the apparent presence of both neuronal and astroglial pathologies concentrated at the depths of cortical sulci (McKee *et al.*, 2016). These preliminary criteria were generated following standardized qualitative methodologies for consensus reporting in which 10 cases of known CTE with ‘late’ stage pathology were selected from a single archive and reviewed by a panel of neuropathologists blind to the original diagnosis. These known CTE cases were assessed alongside cases of multiple other tauopathies and, based on subjective assessments, a consensus agreed upon a so-called defining pathology of CTE. Recognized limitations of this process are the small number of presumed CTE cases selected, their selection as displaying already defined stereotypical pathology and the subjective nature of assessment and review. Hence, these criteria remain preliminary. In keeping with experiences in wider neurodegenerative disease, there is a need for continual reappraisal and refinement of consensus criteria (Montine *et al.*, 2012, 2016; McKeith *et al.*, 2017).

## Conclusion

Our quantitative assessment considered neocortical p-tau pathologies in 22 cases with varying exposures to TBI and known CTE-NC spanning ‘low’- and ‘high’-stage pathology. Furthermore, we compared these pathologies with those observed in material from patients with clinically diagnosed Alzheimer’s disease and confirmed Alzheimer’s disease neuropathologic changes. Our observations suggest that, contrary to the description of the pathognomonic pathology incorporated into current consensus criteria for the neuropathologic assessment and diagnosis of CTE, the distribution of p-tau immunoreactive astroglial pathology, alone, might represent the specific pathology of CTE-NC. As such, current consensus criteria for the identification of CTE-NC might require review and refinement.

## Abbreviations

AD =: Alzheimer’s disease
ADNC =: Alzheimer’s disease neuropathologic changes
ARTAG =: ageing-related tau astrogliopathy
CNDR =: Penn Center for Neurodegenerative Disease Research
CTE-NC =: Chronic traumatic encephalopathy neuropathologic change
NFT =: neurofibrillary tangle
sTBI =: single moderate or severe traumatic brain injury
TBI=: traumatic brain injury
TSA =: thorn-shaped astrocytes
